# Availability of Using Honeybees and Hive Products as Bioindicators of Ambient Pesticide Exposure in Taiwan

**DOI:** 10.3390/toxics12090639

**Published:** 2024-08-31

**Authors:** Chien-Che Hung, Wei-Cheng Chang, Chung-Wen Hsueh, Lih-Ming Yiin

**Affiliations:** Department of Public Health, Tzu Chi University, 701, Sec. 3, Zhongyang Road, Hualien City 970374, Taiwan; gavink23@gms.tcu.edu.tw (C.-C.H.);

**Keywords:** bee pollen, beeswax, bioindicators, dust, honey, honeybees, pesticides

## Abstract

Honeybees and hive products could be used as bioindicators of pesticide exposure in surrounding areas, but the associations have rarely been examined. We collected samples of bees, hive products and environmental dust from 12 apiaries during the blooming season in eastern Taiwan and assessed the relationships between pesticides in apiarian samples and the environment. Samples were analyzed for 14 pesticides using gas or liquid chromatography coupled with mass spectrometry. Sick bees, dead bees, bee pollen, beeswax and environmental dust in the outer rings (>150 m) surrounding the apiaries were contaminated with high levels of pesticides (mean concentration: >270 ng/g in total). In terms of concentrations of all pesticides, insecticides, herbicides and fungicides, most apiarian sample matrices were significantly correlated with environmental dust within a range of 2.5 km (*ρ* > 0.6, *p* < 0.05), suggesting their potential as bioindicators. Of those apiarian matrices with high contamination contents, dead bees or beeswax may be a good bioindicator for all pesticides but not for herbicides, because of the insignificant correlation with environmental dust (*ρ* < 0.5). For all types of pesticides, we recommend sick bees and bee pollen as choices for bioindicators, because of their high contamination levels for detection and complete representativeness of the environment.

## 1. Introduction

Honeybees (*Apis mellifera*) as pollinators in the ecosystem can be easily exposed to environmental pollutants while pollinating. When they return to hives carrying contaminated nectar and pollen, cross-contamination of hive products may occur (e.g., bee pollen, beeswax, honey). Because of possessing the characteristics of sensitivity, ubiquity and stability, honeybees and hive products are considered to be excellent bioindicators for delivering information regarding environmental pollutants in a territory of interest [1,2,3]. Due to their harmful effects and ecological impacts, one pollutant of concern is pesticides, which are usually spread by human activities for various purposes [4]. As pesticides are commonly applied on or near melliferous plants, honeybees and their products appear to be appropriate choices for bioindicators of ambient pesticide exposure.

Honeybees and hive products used as bioindicators of pesticides have been studied extensively. Bees themselves have been used as bioindicators, using counts of dead bees outside hives [1], by chemical analysis of dead or living bees [5,6,7,8] or by a combination of both approaches [9,10], whereas hive products have been examined merely by analysis. Among all types of common hive products, bee pollen is a favorable option for bioindicators because of its easy collection and tendency to be contaminated by pesticides of various types (e.g., fungicide, herbicide, insecticide) [11]. Bee honey is another option [12], and contamination with pesticides is also important due to issues of food safety [13]. Unlike pollen that is raw, honey is a product that is processed through nectar regurgitation by bees and tends to be less contaminated than pollen or bees are [14,15,16]. Beeswax, produced by worker bees, is used to construct honey combs for honey storage as well as larval and pupal protection. Because it is usually long-lasting and lipophilic, beeswax can accumulate liposoluble pesticides brought in by worker bees for a certain period of time [7]. Indeed, pesticides of various types have been found in wax samples by several previous studies [17,18].

Other than honey, pollen and beeswax, royal jelly is also a hive product but rarely seen used as a bioindicator. Royal jelly is a whitish–yellowish jelly-like substance secreted by worker bees and used to feed larvae at the early stage and the queen bee [19]. Pesticides were analyzed and detected in royal jelly by a Greek study [20], suggesting the potential to be a bioindicator for pesticide exposure. Moreover, bee broods in the hives, which are fed on possibly contaminated pollen/beebread and exposed to contaminated beeswax [21,22], could become an option for bioindicators. Thus, these apiarian matrices can all be used as bioindicators of ambient pesticide contamination; however, they may deliver diverse information regarding pesticide exposure due to the different characteristics of pesticides.

Among many papers using various apiarian matrices as bioindicators, few set out to verify the relationships between these matrices and environmental pollutants. Our previous study was dedicated to this work, finding that honeybees could be a good bioindicator of herbicide and fungicide exposures within a range of 1 km [16]. The present work extends the study targets for bioindicators in hive products in addition to bees and further verifies the relationships between apiarian matrices (i.e., healthy bees, sick bees, dead bees, broods, pollen, beeswax honey, royal jelly) and ambient contamination of pesticides (insecticides, herbicides, fungicides). We are convinced that apiarian matrices could become more precise and persuasive bioindicators thereafter.

## 2. Materials and Methods

### 2.1. Participating Apiaries and Sample Collection

**Figure 1:**
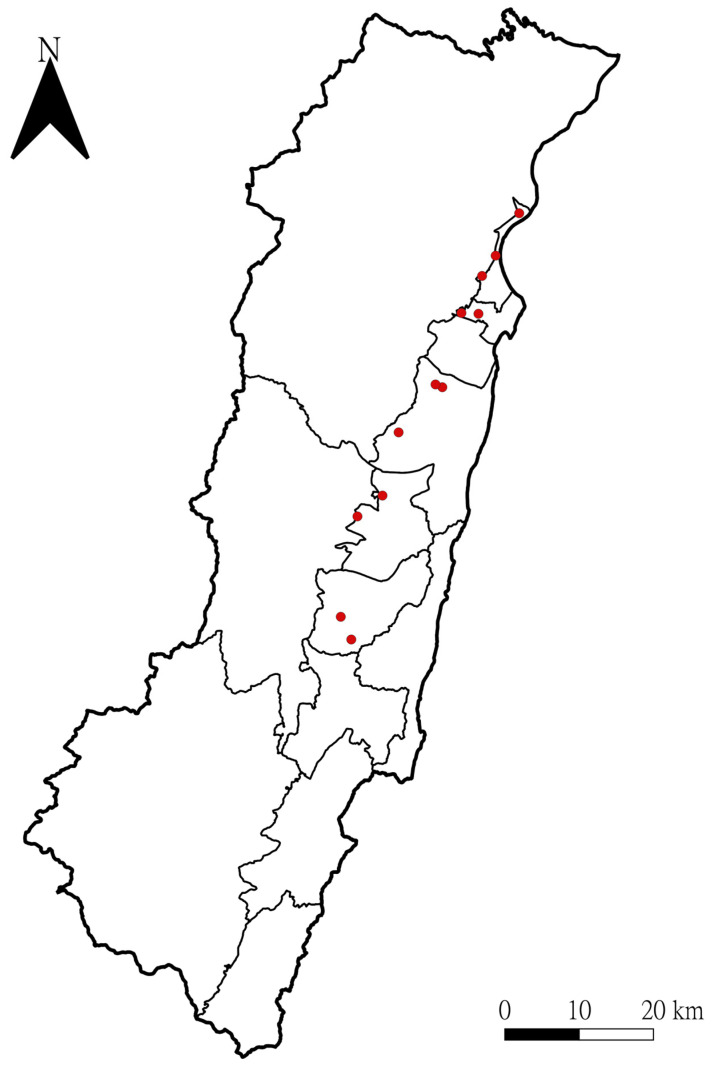
Locations of the 12 apiaries in eastern Taiwan.

This study was a continuous work following the previous study [16], and the 12 participating apiaries plus one as a blank control were similarly located among rural areas of east Taiwan (Figure 1). The apiaries could be stationary or mobile, depending on where and when melliferous plants bloomed. All samples in the study were collected 3–4 times a month during the blooming season for pollination (May to September) in 2022 or 2023. Bees were sampled from approximately one-tenth of the hive boxes at each apiary; healthy bees and broods were collected from inside the boxes, whereas sick and dead bees were taken from outside the boxes. Hive products were taken as convenience samples; beeswax and honey were sampled by the study crew, while pollen and royal jelly were provided by the beekeepers. Environmental dust was collected from the rings around each apiary with different radii (0–50 m, 50–150 m, 150–500 m, 0.5–1.5 km, 1.5–2.5 km); upon availability, 2–4 dust samples were collected from each ring. Location coordinates of the apiaries and environmental dust sampling were determined by a handheld global positioning system navigator (GPSMAP 60CSx, Garmin^®^, New Taipei City, Taiwan), and we used the location data to ensure that environmental samples were collected evenly from each ring around every apiary. Other details of the sampling methods were described previously [16].

### 2.2. Sample Treatment and Analysis

Based on previous experience and recent sale records of pesticides, we updated our list and selected 14 pesticides that are commonly used in Taiwan, including six insecticides, five herbicides, and three fungicides. These were acetamiprid, chlorpyrifos, cypermethrin, dinotefuran, fipronil and indoxacarb for insecticides; ametryn, glyphosate, oxadiazon, paraquat and pendimethalin for herbicides; and dimethomorph, famoxadone and tebuconazole for fungicides. Pesticide standards and solvents for sample processing were purchased either from Sigma-Aldrich^®^ (St. Louis, MO, USA), a branch of Merck KGaA, or Merck itself (Darmstadt, Germany).

The treatment and analysis of bee and honey samples were as same as described in the previous study [16]; broods and beeswax were treated and analyzed in the same way as bee samples, whereas pollen and royal jelly were processed similarly following the approach of honey analysis. In short, weighed and homogenized bee, brood and beeswax samples were added to ethyl acetate for 30-min ultrasonic extraction. The mixture was centrifuged for 10 min, and the supernatant was concentrated, reconstituted with 500 μL n-hexane and filtered for analysis. As for honey, pollen and royal jelly, sample dilution with deionized water (honey, royal jelly) or ethanol aqueous solution (pollen) was necessary. The diluted solutions were processed for solid phase extraction with C18 cartridges (Strata C18 SPE cartridge, Phenomenex, Torrance, CA, USA), which were subsequently washed with deionized water, dried with flushing nitrogen and eluted with n-hexane. The eluates were concentrated and solvent-reconstituted for chromatographic analysis.

Chromatographic analysis using gas chromatography–mass spectrometry (GC-MS, Agilent 6890/5973, Agilent Technologies, Santa Clara, CA, USA) or liquid chromatography–mass spectrometry (LC-MS, Agilent 1200/6460A, Agilent Technologies, Santa Clara, CA, USA) was performed in the same way as reported in the previous study [16]. Briefly, the GC-MS system was equipped with a capillary column (HP-5MS column, 30 m × 0.25 mm × 0.25 µm, Agilent Technologies, Santa Clara, CA USA), with ultra-purity helium (99.9995%) being used as the mobile phase. A temperature profile increasing from 60 °C to 300 °C was set for separation throughout a run. The LC-MS instrument was equipped with a C18 column (Agilent Poroshell 120 EC-C18, 3.0 mm × 100 mm, 2.7 μm, Agilent Technologies, Santa Clara, CA, USA), and deionized water and methanol containing 10 mM ammonium acetate were used as eluents for gradient separation. Any liquid waste generated from the analytic process was stored in waste containers, which were then handled by the waste management department. We followed the guidance published by the U.S. Environmental Protection Agency for quality assurance and control [23]. Either analytic system resulted in the limit of detection of 0.1 ng/g or lower for each target pesticide. The recovery rates measured by spiking samples with standards were around 95%, and the coefficients of variance were all under 10%. The chromatographic results and other information for target pesticides are listed in Table 1.

### 2.3. Data Management and Statistical Analysis

Descriptive statistics of samples analyzed for each pesticide were recorded; concentrations of samples collected from each apiary during the blooming season were categorized by sample matrix and pesticide type and were averaged to represent seasonal concentration profiles. Because samples were not subject to detection, we summarized the sample concentrations within the same pesticide types (i.e., insecticide, herbicide, fungicide) for each sample matrix to facilitate further analyses. Non-detects were replaced with halves of LODs in the data analysis. Analysis of variance (ANOVA) was performed to examine the differences in concentration among the sample matrices within the groups of bees, hive products or environmental dust. Spearman’s correlation analysis was conducted to assess the associations between pesticide concentrations in all kinds of samples of bees, hive products and environmental dust. We used SPSS statistical software package version 23.0 (SPSS Inc., Chicago, IL, USA, 2015) for all statistical analyses.

## 3. Results

**Figure 2:**
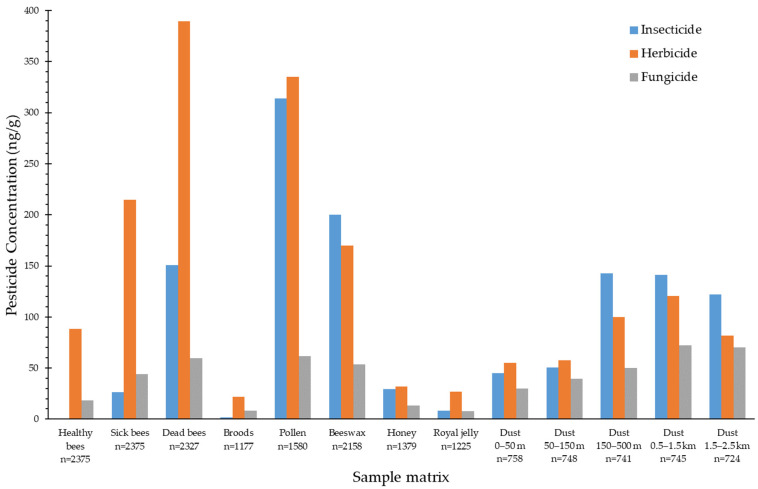
Mean pesticide concentrations by sample matrix.

Healthy, sick and dead bees were individually sampled from the 12 apiaries more than 2300 times, while broods and hive products were collected relatively less often. Environmental dust samples collected from each of the five rings were roughly 750 for the 12 apiaries. Sick bees, dead bees, pollen, beeswax and environmental dust in the outer rings (150–500 m and beyond) possessed higher pesticide contents than other sample matrices did, with the mean concentrations in total pesticides of 270 ng/g or above (Figure 2). The most frequently detected insecticides from all sample matrices were acetamiprid and dinotefuran, whereas herbicides with the highest detection rates were ametryn and oxadiazon. The detection of the three selected fungicides was similarly frequent to one another (Tables S1–S3). Of the pesticides selected for analysis, cypermethrin appeared to be the least detected pesticide consistently throughout all sample matrices, ranging from non-detect (healthy bees) to 23.2% (dust, 1.5–2.5 km).

Among the four bee sample matrices, dead bees were the most contaminated in terms of detection frequency or content of pesticides, followed by sick bees, healthy bees and broods (Table S1, Figure 2). ANOVA indicated that the pesticide concentration differences were significant for comparisons between any pair of two bee sample matrices (*p* < 0.001), except that between healthy bees and broods. All 14 selected pesticides could be found from dead bees with the detection frequencies and the mean concentrations ranging from 30% to 60% and from 20 to 101 ng/g, respectively. Little was detected from broods, and the detection rates and mean concentrations were only up to 28.2% (dimethomorph) and 7.01 ng/g (pendimethalin), respectively. Healthy bees did not carry many insecticides, but were found contaminated with herbicides and fungicides with detection frequencies being roughly between 30% and 50% and the highest mean concentration being 20.7 ng/g (oxadiazon). Sick bees were rich in pesticides, and the contamination level was apparently comparable in of dead bees and healthy bees with any pesticide type.

As for hive products, pollen was the sample matrix loaded with the highest pesticide contents, which were significantly more than that in beeswax in any pesticide type (*p* < 0.001). Most of the detection frequencies for these two matrices were 40% or above, and the mean concentrations ranged from 16.2 to 80.0 ng/g (Table S2). Honey and royal jelly contained significantly lower levels of pesticides than pollen or beeswax did (*p* < 0.001), with the most detection frequencies and mean concentrations under 40% and 10 ng/g, respectively; the concentration difference between both matrices was not significant. Environmental dust contained various levels of pesticides, and seemingly the detection rates and mean concentrations increased as the distances from the apiaries increased (Table S3). For dust in the inner rings (<150 m), the highest detection frequency was approximately 45% and the highest mean concentration was around 14.0 ng/g; for dust in the outer ranges (150 m–2.5 km), the highest detection rate was near 55% and the mean concentrations could be over 30 ng/g. The difference in pesticide concentration between the inner and outer rings was statistically significant (*p* < 0.001).

In terms of all pesticides (sum of insecticides, herbicides and fungicides), healthy bees, sick bees, dead bees, pollen and beeswax were highly correlated with one another (*ρ* > 0.8, *p* < 0.01); their correlations with environmental dust in certain ranges were mostly good or fair (Table 2), indicating the excellent characteristics for bioindicator choices. Broods, honey and royal jelly were correlated with one another (*ρ* > 0.67, *p* < 0.05), but nearly uncorrelated with adult bees (healthy, sick or dead), pollen or beeswax; their correlations with environmental dust were generally poor, except honey’s. None of the associations with environmental dust in the various rings were significant except those of dust in 50–150 m with dust in 0–50 m and 1.5–2.5 km (*ρ* ≥ 0.65, *p* < 0.05). As for insecticides, healthy bees were not associated with any others except broods. The other bee and hive product samples were moderately correlated with one another, and also with environmental dust in certain ranges (Table 2). The correlations among the dust variables were only significant for those between 0–50 m and 50–150 m, and between 0.5–1.5 km and 1.5–2.5 km (*ρ* > 0.7, *p* < 0.01).

For herbicides, healthy bees, sick bees and dead bees were strongly related to one another, but were not associated with beeswax, honey or royal jelly; all apiarian samples except dead bees and beeswax were correlated with environmental dust within the territory of 150 m (Table 3). No correlation was observed beyond the range, suggesting the limit of bioindicator for herbicides. Two dust variable correlations were significant, which occurred between 0–50 m and 50–150 m and between 150–500 m and 0.5–1.5 km (*ρ* > 0.8, *p* < 0.01). In contrast to herbicides, fungicides were the pesticide type with the most significant correlations and appeared almost everywhere among the apiarian samples, and between those and environmental dust in various ranges. In summary, based on the correlation results, any bee or hive product can be used as bioindicators of ambient pesticide exposure to a certain extent, but the detection rates may be a key factor for practical application. The availability, however, may vary with the types of pesticides.

## 4. Discussion

Healthy bees were contaminated with few pesticides, as seen in our previous study [16]; newly sampled broods for this study were also slightly contaminated. Sick and dead bees were processed separately, and it was found that dead bees carried significantly higher concentrations than sick bees did for any pesticide type, especially insecticides. The various concentration profiles shown by healthy, sick and dead bees indicate that health status is a result of exposure to environmental pesticides. It is not surprising to observe relatively high exposures to insecticides in dead bees, because insecticides are meant to be lethal. Elevated levels of herbicides and fungicides in dead bees, however, were also discovered, suggesting that bees’ deaths could be partly attributed to these two types of pesticides, which are less lethal than insecticides. Research has indicated that pesticide mixtures may have synergistic effects on toxicity to bees [24,25], which could explain the relatively high levels of herbicides and fungicides in dead bees. Broods in the hives do not have direct contact with external contaminants and may not have the chance to contact sick or dead bees, because sick bees are usually expelled from the hive boxes and dead bees do not return home. Their exposure sources may be limited to food (e.g., pollen), which is usually consumed in relatively small quantities compared to the body weight in the dose calculation; thus, the pesticide concentrations in broods would be lower than those in food, as seen in Figure 2. Sick and dead bees appear to be better choices for bioindicators than healthy bees and broods because of their elevated levels of pesticides, which are good for detection.

Among hive products, bee pollen was the most pesticide-contaminated sample matrix, followed by beeswax, honey and royal jelly. Pollen tends to be contaminated with pesticides in the field; when brought back by honeybees, it becomes a contamination source of pesticides in the hives. Beeswax is likely cross-contaminated with pesticides from honeybees over time when they transfer pesticide residues from the field to beeswax in the hives during their routine activities. Our findings of pollen and beeswax being the most contaminated hive products are consistent with several previous studies. A Spanish study assessing beehive exposure to pesticides indicated beeswax was the most contaminated hive product, with a high hazard score, and pollen was considered relevant to hazard levels despite its slightly lower pesticide concentrations [26]. An analysis of bee hive products conducted in western Australia found the highest levels of contamination in pollen, followed by that in beebread and beeswax [27]. Based on the previous results and ours, bee pollen and beeswax are certainly rich in pesticides, despite the different mechanisms of contamination; they can be favorable choices for bioindicators, because the elevated levels of contamination provide the sensitivity of being bioindicators. Honey and royal jelly, on the other hand, contained near trace levels of pesticides and were apparently not favored to be bioindicators.

Sick bees, dead bees, bee pollen and beeswax were significantly correlated with the surrounding environment in terms of all pesticides, insecticides, herbicides or fungicides, except dead bees and beeswax for herbicides. The insignificant correlation may be due to the polarity of certain herbicides. Unlike the other herbicides of low polarity, glyphosate and paraquat are of high polarity and water soluble, and their residues in the environment could be washed away by rain, which is quite frequent during the blooming season in eastern Taiwan. The detection rates of glyphosate and paraquat were mostly lower than 40% in environmental dust, compared to 40~55% for the other herbicides, whereas in dead bees, glyphosate and paraquat were detected at frequencies close to those of the other herbicides, suggesting that rain could reduce levels of those water-soluble herbicides in dust and thus result in the low detection rates (Table S3). Moreover, beeswax is lipophilic and not suitable for storing hydrophilic substances, such as glyphosate and paraquat, whose detection rates in beeswax were also relatively low (<40%). Furthermore, unlike insecticides or fungicides spread on plants, herbicides are commonly applied at ground level, which is rarely a routine site for pollination, and honeybees’ contact with herbicides may be relatively slight. With the variability of herbicide concentrations in matrices, it is reasonable that the herbicide correlations between the sample matrices and environmental dust are not likely to be significant.

Pesticide levels in dust were relatively low in the two rings closest to the apiaries (0–150 m), indicating little contamination with pesticides in the neighborhood. This is reasonable because beekeepers would ascertain the surrounding environment to be unpolluted for the safety of their honeybees and hive products. The significant correlations among dust variables of various rings for insecticides and herbicides were few (two for each type), suggesting different sources across the ranges. As for fungicides, however, there were five correlations among the dust variables and multiple ones between apiarian matrices and dust, suggesting that fungicides could be widely spread over the area. Further studies are necessary to understand the information regarding plants, crops and applications of pesticides in the area.

It is worth noting that paraquat, banned for use in Taiwan since 2020, was still detected in all sample matrices, suggesting illegal use of the pesticide in the field. A rough comparison between the results of our previous and present studies indicates that the detection frequencies of paraquat have decreased from 52% to less than 40% [16]. We would like to take this finding as a sign that paraquat is being phased out; nevertheless, a better management for banning paraquat use is necessary.

This study possesses certain strengths. As few studies have explored the associations between bioindicators of and environmental exposure to pesticides previously, we successfully collected all possible apiarian and environmental dust samples to confirm the availability of bioindicators of ambient pesticide exposure, and to distinguish the pesticide contamination levels among these bioindicators. Because one-time apiarian sampling is hardly indicative of environmental exposure [28], we averaged the sample data collected during the blooming season to represent the seasonal profile for each apiary, which served the need for correlation analysis to generate the meaningful results. In contrast, limitations were observed as well. There must have been other pesticides used in the field than the ones selected for analysis, as we occasionally discovered unknown peaks in the chromatogram and found empty bottles of unfamiliar pesticides deserted in the field. Despite the limitations in analysis, we believe that information should be collected sufficiently, with the selected pesticides meeting the requirements of this study. Moreover, as averaging the data for seasonal profiles is good for representativeness of pesticide exposure on one hand, it becomes a shortage on the other. There were only 12 apiaries as data points, which is too few for regression analysis. With such a limitation, we were not able to obtain further specific information regarding the associations between apiarian matrices and the environment surrounding the apiaries, but used the correlation results instead.

## 5. Conclusions

This study confirmed that honeybees and hive products could serve as bioindicators for ambient pesticide exposure to a certain extent, by showing several significant associations with the surrounding environment within 2.5 km. Sample matrices with high contamination levels are favorable because of the good detection rates for practical use. Of the matrices with high contamination, dead bees and beeswax are indicative of ambient exposure to insecticides and fungicides, but not herbicides. Sick bees and bee pollen are even better choices, because of their good correlations with the environment and all types of pesticides (i.e., insecticides, herbicides, fungicides).

## Figures and Tables

**Table 1:** General information regarding target pesticides.

Type and Name	Analytical Method	Retention Time (min)	Main Use (Agriculture, Environmental Sanitation, or Both)
Insecticide			
Acetamiprid	LC-MS	2.31	Agriculture
Chlorpyrifos	GC-MS	8.62	Both
Cypermethrin	GC-MS	22.71	Agriculture
Dinotefuran	LC-MS	6.41	Both
Fipronil	GC-MS	6.31	Both
Indoxacarb	LC-MS	3.36	Agriculture
Herbicide			
Ametryn	LC-MS	4.51	Agriculture
Glyphosate	LC-MS	1.13	Agriculture
Oxadiazon	LC-MS	8.81	Agriculture
Paraquat	LC-MS	3.29	Agriculture
Pendimethalin	LC-MS	2.79	Agriculture
Fungicide			
Dimethomorph	GC-MS	15.45	Agriculture
Famoxadone	LC-MS	6.75	Agriculture
Tebuconazole	LC-MS	6.49	Agriculture

**Table 2:** Spearman’s correlation coefficients among sample matrices for all pesticides and insecticides (n = 12).

	All	Healthy Bees	Sick Bees	Dead Bees	Broods	Pollen	Beeswax	Honey	Royal Jelly	Dust 0–50 m	Dust 50–150 m	Dust 150–500 m	Dust 0.5–1.5 km	Dust 1.5–2.5 km
Insect.														
**Healthy bees**			0.909 **	0.832 **	0.385	0.776 **	0.895 **	0.622 *	0.510	0.559	0.839 **	0.319	0.469	0.839 **
**Sick bees**		0.394		0.867 **	0.336	0.874 **	0.853 **	0.462	0.566	0.538	0.734 **	0.238	0.273	0.671 *
**Dead bees**		0.480	0.704 *		0.077	0.734 **	0.839 **	0.455	0.371	0.455	0.811 **	0.490	0.364	0.671 *
**Broods**		0.631 *	0.708 **	0.624 *		0.329	0.182	0.671 *	0.755 **	0.350	0.294	0.186	−0.119	0.224
**Pollen**		0.393	0.396	0.706 *	0.653 *		0.853 **	0.448	0.566	0.741 **	0.664 *	0.287	0.287	0.364
**Beeswax**		0.393	0.623 *	0.790 **	0.641 *	0.942 **		0.469	0.273	0.538	0.769 **	0.259	0.434	0.615 *
**Honey**		0.480	0.708 *	0.734 **	0.650 *	0.517	0.601 *		0.699 *	0.483	0.643 *	0.711 **	0.503	0.594 *
**Royal jelly**		0.498	0.574	0.761 **	0.774 **	0.805 **	0.769 **	0.471		0.531	0.441	0.480	0.224	0.343
**Dust 0–50 m**		−0.044	0.392	0.566	0.386	0.629 *	0.629 *	0.448	0.740 **		0.664 *	0.385	0.259	0.203
**Dust 50–150 m**		0.218	0.778 **	0.853 **	0.624 *	0.545	0.615 *	0.559	0.776 **	0.706 *		0.455	0.476	0.650 *
**Dust 150–500 m**		0.306	0.357	0.406	0.725 **	0.378	0.294	0.35	0.558	0.441	0.469		0.469	0.368
**Dust 0.5–1.5 km**		0.218	0.473	0.434	0.532	0.573	0.483	0.664 *	0.428	0.385	0.294	0.406		0.545
**Dust 1.5–2.5 km**		0.480	0.704 *	0.566	0.486	0.573	0.727 **	0.545	0.609 *	0.378	0.448	0.287	0.727 **	

*: *p* < 0.05; **: *p* < 0.01.

**Table 3:** Spearman’s correlation coefficients among sample matrices for herbicides and fungicides (n = 12).

	Herb.	Healthy Bees	Sick Bees	Dead Bees	Broods	Pollen	Beeswax	Honey	Royal Jelly	Dust 0–50 m	Dust 50–150 m	Dust 150–500 m	Dust 0.5–1.5 km	Dust 1.5–2.5 km
Fung.														
**Healthy bees**			0.888 **	0.713 **	0.531	0.629 *	0.350	0.210	0.455	0.566	0.881 **	0.322	0.056	0.245
**Sick bees**		0.895 **		0.839 **	0.587 *	0.727 **	0.476	0.154	0.497	0.469	0.713 **	0.322	−0.032	0.203
**Dead bees**		0.776 **	0.860 **		0.245	0.580 *	0.573	−0.084	0.315	0.203	0.469	0.490	0.203	0.196
**Broods**		0.814 **	0.860 **	0.796 **		0.882 **	0.787 **	0.784 **	0.680 *	0.462	0.608 *	−0.105	−0.501	0.140
**Pollen**		0.839 **	0.741 **	0.594 *	0.721 **		0.727 **	0.077	0.469	0.664 *	0.615 *	0.280	0.077	0.147
**Beeswax**		0.734 **	0.783 **	0.839 **	0.690 *	0.570		−0.294	0.427	0.322	0.392	0.259	−0.056	0.287
**Honey**		0.881 **	0.846 **	0.811 **	0.550	0.776 **	0.699 *		0.524	0.594 *	0.427	−0.007	−0.011	−0.042
**Royal jelly**		0.690 *	0.620 *	0.400	−0.039	0.560	0.230	0.669 *		0.755 **	0.622 *	0.273	−0.081	−0.007
**Dust 0–50 m**		0.470	0.692 *	0.550	0.560	0.490	0.430	0.490	0.420		0.804 **	0.168	−0.032	−0.063
**Dust 50–150 m**		0.430	0.657 *	0.748 **	0.709 **	0.480	0.629 *	0.450	0.250	0.776 **		0.231	−0.039	0.273
**Dust 150–500 m**		0.615 *	0.734 **	0.643 *	0.667 *	0.804 **	0.832 **	0.615 *	0.200	0.550	0.671 *		0.858 **	−0.196
**Dust 0.5–1.5 km**		0.825 **	0.888 **	0.748 **	0.761 **	0.811 **	0.748 **	0.734 **	0.520	0.678 *	0.692 *	0.825 **		−0.158
**Dust 1.5–2.5 km**		0.797 **	0.790 **	0.790 **	0.667 *	0.601 *	0.530	0.916 **	0.577 *	0.440	0.380	0.470	0.685 *	

*: *p* < 0.05; **: *p* < 0.01.

## Data Availability

The data presented in this study are available on request from the corresponding author.

## Supplementary Materials

The following supporting information can be downloaded at: https://www.mdpi.com/article/10.3390/toxics12090639/s1, Table S1: Pesticide concentrations in bees by type; Table S2: Pesticide concentrations in hive products by type; Table S3: Pesticide concentrations in environmental dust by distance.
