# Supplementary material for: Availability of Using Honeybees and Hive Products as Bioindicators of Ambient Pesticide Exposure in Taiwan
Source: Toxics. 2024 Aug 31;12(9):639. doi: 10.3390/toxics12090639 (PMC11435600; doi:10.3390/toxics12090639)
Supplement: Supplementary file 1 [file toxics-12-00639-s001.zip › toxics-3164139-supplementary.pdf]

Table S1. Pesticide concentrations in bees by type.

Sample Type	Pesticide	DF (%)	Mean±SD (ng/g)	50th Percentile (ng/g)	75th Percentile (ng/g)	Maximum (ng/g)
**Healthy bees (n=2375)**	*Insecticides*					
	Acetamiprid	0	LOD	LOD	LOD	LOD
	Chlorpyrifos	3.58	0.29±3.01	LOD	LOD	14.0
	Cypermethrin	0	LOD	LOD	LOD	LOD
	Dinotefuran	0	LOD	LOD	LOD	LOD
	Fipronil	2.32	0.19±1.81	LOD	LOD	8.92
	Indoxacarb	0	LOD	LOD	LOD	LOD
	Herbicides					
	Ametryn	50.4	16.6±41.1	25.1	42.5	177
	Glyphosate	31.3	15.1±28.4	LOD	35.5	117
	Oxadiazon	47.0	20.7±40.8	LOD	60.1	172
	Paraquat	35.1	16.0±41.3	LOD	38.3	200
	Pendimethalin	46.0	19.8±39.7	LOD	42.8	193
	Fungicide					
	Dimethomorph	32.9	7.28±15.3	LOD	22.2	63.2
	Famoxadone	29.2	6.03±13.3	LOD	15.3	62.5
	Tebuconazole	29.2	5.03±20.4	LOD	38.2	50.9
**Sick bees (n=2375)**	*Insecticides*					
	Acetamiprid	37.2	5.43±14.2	LOD	23.3	51.2
	Chlorpyrifos	23.1	4.63±10.9	LOD	LOD	55.6
	Cypermethrin	8.59	3.15±11.4	LOD	LOD	56.0
	Dinotefuran	24.8	3.09±7.53	LOD	LOD	38.8
	Fipronil	22.8	5.97±19.8	LOD	LOD	107
	Indoxacarb	18.3	4.09±14.3	LOD	LOD	81.7
	Herbicides					
	Ametryn	56.4	49.9±96.6	86.8	114	406
	Glyphosate	39.3	41.0±107	LOD	108	480
	Oxadiazon	56.5	48.8±123	101	121	582
	Paraquat	45.0	42.7±87.3	LOD	89.7	402
	Pendimethalin	50.1	32.2±69.3	51.1	82.2	297
	Fungicide					
	Dimethomorph	37.1	15.9±37.7	LOD	40.7	165
	Famoxadone	46.7	14.8±29.9	LOD	33.2	129
	Tebuconazole	41.5	13.3±40.1	LOD	115	153
**Dead bees (n=2327)**	*Insecticides*					
	Acetamiprid	45.8	28.8±39.7	LOD	63.2	148
	Chlorpyrifos	30.7	28.6±71.2	LOD	118	483
	Cypermethrin	20.4	19.6±58.5	LOD	LOD	396
	Dinotefuran	33.2	16.6±69.3	LOD	106	265
	Fipronil	42.8	43.6±125	LOD	135	684
	Indoxacarb	32.4	13.3±37.5	LOD	35.5	200
	Herbicides					
	Ametryn	60.5	101±193	137.52	177	1003
	Glyphosate	47.6	72.7±106	LOD	173	593
	Oxadiazon	59.2	84.2±143	174.7	204	703
	Paraquat	50.3	80.2±144	143.25	203	631
	Pendimethalin	53.7	50.6±96.9	81.71	116	404
	Fungicide					
	Dimethomorph	45.2	18.2±40.3	LOD	45.3	183
	Famoxadone	51.8	21.5±37.9	40.65	52.6	145
	Tebuconazole	42.2	19.6±66.3	LOD	141	188

	Insecticides					
	Acetamiprid	3.40	0.23±1.42	LOD	LOD	6.74
	Chlorpyrifos	12.2	0.73±2.88	LOD	LOD	14.8
	Cypermethrin	0	LOD	LOD	LOD	LOD
	Dinotefuran	0	LOD	LOD	LOD	LOD
	Fipronil	6.29	0.50±2.79	LOD	LOD	14.7
	Indoxacarb	0	LOD	LOD	LOD	LOD
	Herbicides					
**Broods (n=1177)**	Ametryn	19.4	3.33±9.25	LOD	LOD	56.7
	Glyphosate	11.3	2.06±9.03	LOD	LOD	42.3
	Oxadiazon	26.7	5.85±17.5	LOD	15.3	79.4
	Paraquat	11.8	3.31±7.25	LOD	LOD	38.5
	Pendimethalin	31.4	7.01±15.3	LOD	12.3	79.8
	*Fungicid*e					
	Dimethomorph	28.2	4.06±10.7	LOD	10.1	58.4
	Famoxadone	12.1	1.55±4.91	LOD	LOD	27.0
	Tebuconazole	21.4	2.56±8.32	LOD	LOD	45.5

DF: Detection frequency; LOD: Limit of detection; SD: Standard deviation.

Table S2. Pesticide concentrations in hive products by type.

Sample Type	Pesticide	DF (%)	Mean±SD (ng/g)	50th Percentile (ng/g)	75th Percentile (ng/g)	Maximum (ng/g)
**Pollen**	*Insecticides*					
**(n=1580)**	Acetamiprid	56.7	62.7±88.8	89.7	144	378
	Chlorpyrifos	47.8	70.1±154	LOD	253	787
	Cypermethrin	38.4	27±55.3	LOD	84.1	256
	Dinotefuran	54.7	29.2±53.3	75.5	106	270
	Fipronil	51.4	76.9±175	153	354	703
	Indoxacarb	40.6	48.3±195	LOD	368	807
	Herbicides					
	Ametryn	64.0	72.2±121	100	155	532
	Glyphosate	51.0	69.3±134	128	162	587
	Oxadiazon	53.1	62.5±175	130	158	758
	Paraquat	53.4	80±104	122	189	506
	Pendimethalin	55.6	50.9±67.4	92.6	121.3	309
	Fungicide					
	Dimethomorph	41.5	20.7±34.2	LOD	53.2	178
	Famoxadone	53.1	19.8±37.3	35.5	41.2	197
	Tebuconazole	44.1	20.9±72.1	LOD	178	236
**Beeswax**	*Insecticides*					
**(n=2158)**	Acetamiprid	51.4	48.3±54.7	68.3	90.7	322
	Chlorpyrifos	37.5	34.6±90.9	LOD	109	471
	Cypermethrin	23.6	20.0±52.6	LOD	LOD	257
	Dinotefuran	47.3	20.5±80.1	LOD	128	312
	Fipronil	40.1	56.0±144	LOD	172	651
	Indoxacarb	35.7	21.0±54.2	LOD	55.2	299
	Herbicides					
	Ametryn	54.8	35.2±43.4	55.4	91.4	211
	Glyphosate	39.2	39.3±63.7	LOD	91.5	300
	Oxadiazon	50.1	34.6±55.9	71.8	98.5	254
	Paraquat	39.9	32.9±31.6	LOD	57.9	165
	Pendimethalin	46.6	28.0±33.7	LOD	68.8	179
	Fungicide					
	Dimethomorph	46.0	19.1±27.9	LOD	47.7	153
	Famoxadone	48.1	16.2±33.9	LOD	30.1	142
	Tebuconazole	39.8	18.0±57.8	LOD	141	188
**Honey**	*Insecticides*					
**(n=1379)**	Acetamiprid	37.6	5.85±17.8	LOD	10.6	73.3
	Chlorpyrifos	28.5	5.89±19.2	LOD	27.9	115
	Cypermethrin	4.28	1.91±5.02	LOD	LOD	24.7
	Dinotefuran	18.1	3.18±19.3	LOD	LOD	91.3
	Fipronil	29.3	8.32±30.2	LOD	58.9	103
	Indoxacarb	22.6	4.07±17.3	LOD	LOD	77.4
	Herbicides					
	Ametryn	39.2	4.85±10.5	LOD	8.23	64.5
	Glyphosate	17.9	6.07±11.3	LOD	LOD	59.3
	Oxadiazon	34.6	6.80±10.7	LOD	15.5	63.1
	Paraquat	12.6	3.44±8.51	LOD	LOD	35.3
	Pendimethalin	41.6	10.6±19.1	LOD	23.6	95.1
	Fungicide					
	Dimethomorph	37.1	5.67±10.7	LOD	15.2	63.5
	Famoxadone	16.1	2.98±14.5	LOD	LOD	61.8
	Tebuconazole	29.9	4.71±15.6	LOD	37.1	49.4
**Royal Jelly**	*Insecticides*					
**(n=1225)**	Acetamiprid	3.7	0.93±11.0	LOD	LOD	50.0

Chlorpyrifos	18.1	2.68±8.38	LOD	LOD	34.4
Cypermethrin	2.12	0.79±6.07	LOD	LOD	23.8
Dinotefuran	4.90	0.22±2.15	LOD	LOD	12.1
Fipronil	13.9	3.16±15.9	LOD	LOD	74.9
Indoxacarb	3.76	0.49±2.81	LOD	LOD	15.1
Herbicides					
Ametryn	29.4	5.21±14.2	LOD	8.84	66.8
Glyphosate	14.8	3.97±5.01	LOD	LOD	24.7
Oxadiazon	36.5	7.03±12.04	LOD	14.3	64.2
Paraquat	9.31	2.6±5.29	LOD	LOD	30.1
Pendimethalin	42.0	8.02±12.7	LOD	15.5	61.4
*Fungicid*e					
Dimethomorph	21.1	3.72±14.0	LOD	LOD	76.0
Famoxadone	8.90	1.34±7.88	LOD	LOD	42.3
Tebuconazole	17.4	2.51±9.52	LOD	LOD	42.5

DF: Detection frequency; LOD: Limit of detection; SD: Standard deviation.

Table S3. Pesticide concentrations in environmental dust by distance.

Sample Location	Pesticide	DF (%)	Mean±SD (ng/g)	50th Percentile (ng/g)	75th Percentile (ng/g)	Maximum (ng/g)
**0 – 50 m**	*Insecticides*					
**(n=758)**	Acetamiprid	27.0	6.06±32.2	LOD	41.3	67.9
	Chlorpyrifos	25.7	10.2±30.2	LOD	32.3	119
	Cypermethrin	13.7	5.49±17.1	LOD	LOD	72.9
	Dinotefuran	32.5	7.28±38.8	LOD	67.9	122
	Fipronil	33.9	9.98±25.8	LOD	35.5	79.9
	Indoxacarb	22.6	6.16±17.7	LOD	LOD	61.1
	Herbicides					
	Ametryn	47.2	12.7±29.4	LOD	21.5	111
	Glyphosate	35.6	10.2±30.7	LOD	24.2	126
	Oxadiazon	40.5	13.7±28.7	LOD	31.5	105
	Paraquat	39.1	8.45±20.2	LOD	17.7	62.7
	Pendimethalin	43.1	10.2±28.9	LOD	22.3	118
	Fungicide					
	Dimethomorph	39.2	10.6±29.8	LOD	25.5	133
	Famoxadone	43.4	9.03±22.0	LOD	16.2	93.4
	Tebuconazole	42.1	10.1±49.7	LOD	96.7	129
**50 – 150 m**	*Insecticides*					
**(n=748)**	Acetamiprid	44.3	8.03±16.4	LOD	19.4	56.3
	Chlorpyrifos	41.0	11.1±26.2	LOD	34.5	85.9
	Cypermethrin	20.7	7.23±10.5	LOD	LOD	44.6
	Dinotefuran	41.8	7.93±33.9	LOD	57.7	79.4
	Fipronil	33.3	8.57±24.6	LOD	27.6	72.4
	Indoxacarb	26.6	7.82±14.2	LOD	19.5	46.0
	Herbicides					
	Ametryn	44.8	11.1±21.6	LOD	18.8	114
	Glyphosate	32.5	10.6±21.2	LOD	21.2	109
	Oxadiazon	44.8	10.6±28.5	LOD	21.9	122
	Paraquat	36.2	12.5±70.7	LOD	29.1	637
	Pendimethalin	43.1	12.8±40.9	LOD	20.7	188
	Fungicide					
	Dimethomorph	44.0	13.1±20.8	LOD	21.3	187
	Famoxadone	42.5	12.8±22.1	LOD	27.3	99.3
	Tebuconazole	43.1	13.5±63.3	LOD	120	159
**150 – 500 m**	*Insecticides*					
**(n=741)**	Acetamiprid	44.1	23.9±30.9	LOD	63.2	153
	Chlorpyrifos	42.9	28.5±79.6	LOD	106	413
	Cypermethrin	21.9	20.9±27.8	LOD	LOD	165
	Dinotefuran	53.9	27.6±60.1	79.7	105	186
	Fipronil	34.6	23.1±40.5	LOD	60.8	165
	Indoxacarb	29.7	18.6±31.7	LOD	39.7	160
	Herbicides					
	Ametryn	54.4	21±40.2	34.9	44.6	200
	Glyphosate	36.6	14.8±30.6	LOD	39.4	109
	Oxadiazon	46.3	20.8±41.9	LOD	52.8	158
	Paraquat	36.8	19.3±28.9	LOD	33.3	97.6
	Pendimethalin	45.9	24.3±41.9	LOD	57.1	190
	Fungicide					

	Dimethomorph	43.7	16.2±34.9	LOD	35.2	163
	Famoxadone	52.2	16.4±30.2	25.5	37.6	107
	Tebuconazole	47.1	17.4±73.3	LOD	159	211
**500 – 1500 m (n=745)**	*Insecticides*					
	Acetamiprid	47.5	26.3±50.8	LOD	55.3	252
	Chlorpyrifos	39.3	25.7±52.5	LOD	78.9	151
	Cypermethrin	20.1	21.2±65.6	LOD	LOD	134
	Dinotefuran	51.0	19.9±71.0	50.1	151	249
	Fipronil	41.1	30.1±53.0	LOD	94.3	180
	Indoxacarb	37.3	18.1±29.7	LOD	41.2	113
	Herbicides					
	Ametryn	54.2	25.2±45.4	44.0	68.0	193
	Glyphosate	41.3	20±41.0	LOD	47.0	182
	Oxadiazon	46.6	23.1±39.1	LOD	59.0	122
	Paraquat	39.7	23.7±35.4	LOD	47.5	129
	Pendimethalin	49.1	28.5±59.5	LOD	70.8	212
	Fungicide					
	Dimethomorph	43.5	23.2±53.6	LOD	50.6	196
	Famoxadone	51.7	23.6±43.3	39.9	50.1	167
	Tebuconazole	44.8	25.5±92.5	LOD	213	283
**1500 – 2500 m (n=745)**	*Insecticides*					
	Acetamiprid	50.4	18.6±41.5	32.3	40.1	142
	Chlorpyrifos	40.5	32.1±76.3	LOD	109	493
	Cypermethrin	23.2	17.6±21.1	LOD	LOD	120
	Dinotefuran	47.7	20.1±79.2	LOD	151	304
	Fipronil	36.3	18.0±43.2	LOD	59.6	219
	Indoxacarb	25.7	15.7±26.9	LOD	40.1	149
	Herbicides					
	Ametryn	54.3	19.1±27.9	25.9	40.3	101
	Glyphosate	35.9	13.3±30.1	LOD	39.6	125
	Oxadiazon	47.8	16.0±27.2	LOD	25.2	126
	Paraquat	30.3	12.7±16.9	LOD	21.5	55.0
	Pendimethalin	47.5	20.6±38.0	LOD	54.2	132
	Fungicide					
	Dimethomorph	47.2	25.0±35.2	LOD	70.1	256
	Famoxadone	50.4	23.1±40.1	35.5	57.2	184
	Tebuconazole	49.0	22.0±60.7	LOD	128	170

DF: Detection frequency; LOD: Limit of detection; SD: Standard deviation.
